# Inequalities in the prevalence of skilled birth attendance in Ghana between 1993 and 2014

**DOI:** 10.1093/inthealth/ihac071

**Published:** 2022-11-09

**Authors:** Justice Kanor Tetteh, Edward Kwabena Ameyaw, Collins Adu, Ebenezer Agbaglo, Pascal Agbadi, Jerry John Nutor

**Affiliations:** Department of Population and Health, University of Cape Coast, Cape Coast, Ghana; Institute of Policy Studies and School of Graduate Studies, Lingnan University, Hong Kong; Department of Health Promotion, Education and Disability Studies, Kwame Nkrumah University of Science and Technology, Kumasi, Ghana; College of Public Health, Medical and Veterinary Sciences, James Cook University, Townsville, Queensland 4811, Australia; Department of English, University of Cape Coast, Cape Coast, Ghana; Department of Nursing, College of Health Sciences, Kwame Nkrumah University of Science and Technology, Kumasi, Ghana; Department of Family Health Care Nursing, School of Nursing, University of California, San Francisco, 2 Koret Way, Suite N431G, San Francisco, CA 94143, USA

**Keywords:** demographic and health surveys, Ghana, global health, inequality, maternal health, skilled birth attendance

## Abstract

**Background:**

Globally, maternal and neonatal health remains a public health priority, particularly for resource-constrained regions like sub-Saharan Africa (SSA). Skilled birth attendance (SBA) is essential in promoting maternal and neonatal health. This study investigated the inequalities in the prevalence of SBA in Ghana using data from the Ghana Demographic and Health Survey (GDHS) between 1993 and 2014.

**Methods:**

Data were analysed using the World Health Organization's Health Equity Assessment Toolkit software. In analysing the data, we first disaggregated SBA by four inequality stratifiers: wealth index, education, residence, and region. Second, we measured the inequality through summary measures, namely difference, population attributable risk, ratio, and population attributable fraction. A 95% confidence interval was constructed for point estimates to measure statistical significance.

**Results:**

Throughout the period, SBA was highest among women in the highest wealth quintile and those with a secondary or higher level of education. The analysis also indicated that SBA was highly concentrated among urban residents in 1993 (80.78 [95% uncertainty interval {UI} 76.20–84.66]) and persisted to 2014 (91.55 [95% UI 88.80–93.68]). In 1993, Northern region recorded the lowest prevalence of SBA in Ghana (15.69 [95% UI 11.20–21.54]) and the region consistently recorded the lowest SBA prevalence even into 2014 (38.21 [95% UI 27.44–50.27]).

**Conclusions:**

There are significant inequalities in SBA across education, wealth, residence, and region in Ghana. To enhance SBA, there is the need for policymakers and interventionists to design and develop targeted policies and programs that are tailored to the needs of the subpopulations at risk of low SBA: women with no formal education, those within the poorest wealth quintile, rural-dwelling women and women in the Northern region. This will facilitate the uptake of SBA and ultimately translate into the realization of Sustainable Development Goals 3.1 and 3.2.

## Introduction

Globally, maternal and neonatal health remains a public health priority, particularly for resource-constrained regions like sub-Saharan Africa (SSA).^1,2^ Evidence of a global consensus on the importance of maternal health was initially captured in the United Nations’ Millennium Development Goal (MDG) 5, which sought to reduce maternal mortality by three-quarters between the years 1990 and 2015.^3^ Although many investments were made towards achieving the MDG 5, many countries did not meet the target in 2015. Subsequently, the global community signed unto a new set of goals called the Sustainable Development Goals (SDGs) in 2015. SDG 3.1 seeks to reduce the maternal mortality rate (MMR) to <70 deaths per 100 000 live births by 2030.^4^ One of the main indicators for the realization of this goal is a key indicator (SDG 3.1.2) that highlights the proportion of births attended by skilled birth attendants or skilled health personnel.^5^ Other key factors that could help to achieve this goal include the utilization of clean delivery kits to prevent infection during delivery, frequent and prompt antenatal care visits, community mobilization or community-led programs for maternal health and maternal healthcare fee waivers or free health insurance for pregnant women.^6^ This study focuses on skilled birth attendance (SBA) as a key indicator.

In 2004 the World Health Organization (WHO), the International Federation of Gynecology and Obstetrics and the International Confederation of Midwives defined skilled birth attendants and their core functions as accredited health personnel with the requisite proficient training and skills to be able to manage uncomplicated pregnancies, childbirth and the immediate postnatal period as well as identifying, managing and referring complications in women and newborns.^6^ Essentially the presence of skilled birth attendants at birth has been recognized by scholars as a great intervention for averting many preventable maternal and neonatal morbidities and mortalities.^7^ This is reflected in statistics from the United Nations Population Fund^8^ that indicate that countries with ≥80% or higher SBA have lower MMRs (i.e. <200 per 100 000 live births). It is recommended that deliveries should take place in a range of appropriate settings, from home to tertiary referral centres, depending on availability and need.^8^ SBA therefore remains a critical tool for good maternal health outcomes and many resources have been invested to increase its coverage globally.

Globally there has been a significant increase in SBA coverage. For example, during the same period this study's data were collected, SBA coverage increased from 61.5% in 2000 to 73% in 2013.^8^ Despite this increase in global coverage, there are still inequalities in the prevalence and distribution among many low- and middle-income countries (LMICs).^9,10^ Again, despite the importance and benefits associated with SBA, SSA and South Asia are often characterized by a low prevalence of SBA compared with other regions like Europe.^10,11^ Although in SSA there have been some improvements in the prevalence of SBA over the years,^12^ this has been characterized by significant regional and country disparities. In 2014, reports showed that there was only a 13% increase in SBA deliveries within a 25-y period; that is, from 43% in 1990 to 59% in 2014.^5^ In Ethiopia for instance, the percentage of deliveries assisted by skilled birth attendants increased from 5.6% in 2000 to 27.7% in 2016.^13^ Other reports have shown a significantly low prevalence of SBA among other SSA countries, including Niger (32.6% in 2012), Mali (39.9% in 2013) and Sierra Leone (45.2% in 2013).^14^ However, in other SSA countries such as Ghana, the story is different.

In 2008 the Ghana Ministry of Health declared maternal mortality a national emergency and subsequently explored diverse ways of improving access to SBA, which led to the adoption of the free maternal healthcare policy and program for all pregnant women in Ghana.^6,15^ Following this policy direction in Ghana, there have been some improvements in SBA coverage and the 2014 Ghana Demographic and Health Survey (GDHS) shows coverage of 75.81%, up from 44% in 1993.^16^ There have also been reports of potential rural–urban disparities in SBA utilization, with less than half of rural women utilizing SBA compared with about two-thirds of urban women utilizing SBA.^17^ Other studies have found disparities in SBA utilization across the dimensions of education and wealth, where the poor and those with low education utilize SBA the least.^18–20^

Despite these observed disparities and potential inequalities in the prevalence or coverage of skilled birth deliveries in Ghana, our extensive literature search revealed that no study has investigated the magnitude of inequalities in SBA in Ghana over time. This presents a gap in the existing literature on SBA and its role in MMR reduction in Ghana. Therefore the current study seeks to investigate inequalities in the prevalence of births attended by skilled health personnel in Ghana over time using data from the Ghana DHS from 1993 to 2014. The findings of this study will be vital to policymakers in formulating targeted policies and interventions that are tailored to the individual needs of the subpopulations to reduce the inequalities in SBA. This could ultimately expedite the realization of SDG 3.1 and 3.2.

## Methods

### Study area

The present study was conducted in Ghana. Ghana shares boundaries to the north with Burkina Faso, east with Togo, west with Ivory Coast, and south with the Atlantic Ocean. It has a total land area of 238 533 km^2^.^21^ As of 2010, the population and housing census reported a total population of 24 658 823.^22^ Previously the country had 10 administrative regions (Western, Central, Greater Accra, Volta, Eastern, Ashanti, Brong Ahafo, Northern, Upper East and Upper West) but in 2019 they were further divided into 16 administrative regions. Slightly more than half (51%) of the population live in urban areas. In Ghana, the total fertility rate is 4.2 children per woman and childbearing peaks at the age of 25–29 y and decreases sharply after age 39 y.^16^ According to the 2014 Ghana DHS, rural women have about 1.7 more children than urban women and about 74% of births in Ghana are delivered with the assistance of a skilled health professional, 16% by a traditional birth attendant, 7% by a relative or other person and 3% are not assisted by anyone. In the Northern, Upper East, and Upper West regions, traditional birth attendants play important roles in assisting in the delivery of 41% of births.^16^

### Data source and study population

Secondary de-identified data from the GDHS from 1993 to 2014 were used for this study. The first version, 1988, was not considered because some of the variables were not consistent with those from 1993 to 2014. DHSs are conducted by the DHS Program (Rockville, MD, USA) to collect demographic data from over 85 LMICs in about 5-year intervals. These surveys collect data from women, children, men and households. For women, the survey solicits information on fertility, family planning and utilization of maternal health services such as skilled delivery and antenatal and postnatal care. A dual-stage sampling approach is used to select the respondents. For each survey, before sampling, the procedures followed by the recent population and housing census are used to guide the selection of enumeration areas (EAs) that are chosen proportional to the size of the EA with independent selection in each sampling stratum. The EA constitutes the number of residential households inhabiting the delineated area. A household listing operation is carried out in all the selected EAs and the resulting list of households serves as a sampling frame for the selection of households to the second stage. The detailed methodologies employed in the GDHS are included in the final reports such as that of the 2014 GDHS.^16^ In this study, women 15–49 y of age with birth histories 2–3 y before the survey constituted our sample. The datasets contained the following: 2204 participants in 1993, 1925 participants in 1998, 2171 participants in 2003, 1757 participants in 2008 and 3507 participants in 2014.

### Measures of inequality

The main inequality variable measured in this study is SBA. It is measured as the proportion of women whose births were attended by skilled personnel. Skilled personnel include doctors, nurses, midwives, auxiliary midwives and community health nurses/officers.^16^ We disaggregated SBA by four inequality stratifiers: economic status, education, subnational region, and place of residence. Economic status is a proxy variable measured by the wealth index. In the DHS, the wealth index is computed using different assets possessed by the household. These assets are aggregated using the Principal Component Analysis (PCA) technique^22^ and grouped into quantiles (20%). Assets of those in the first category (poorest) fell in the first 20% and those of the highest (richest) fell in the upper 80–100%. The education level was captured by the highest level of education the woman had attained. This was grouped as no education, primary, and secondary/highest and the place of residence was captured as urban or rural. The region was also classified as one of the then 10 administrative regions of Ghana.

### Analytical procedure

WHO's Health Equity Assessment Toolkit (HEAT) software was used for analysing the socio-economic and geographical inequalities associated with SBA.^23^ We followed two principal steps to organize the data and the results. Initially we disaggregated SBA by the four inequality stratifiers: economic status, education status, place of residence and region of residence. This was done to present the estimates of SBA viz-à-viz the inequality stratifiers. Subsequently we presented the absolute difference (D), population attributable risk (PAR) and relative inequality summary measures (population attributable fraction [PAF] and ratio [R]). Previous evidence from the WHO^18^ shows that both absolute and relative summary measures in a single health inequality study are more informative for policy formulation. Detailed procedures and calculations of the summary measures have been reported elsewhere.^23^

The PAF and PAR assume positive values for favourable health intervention indicators and negative values for adverse health outcome indicators. Zero shows the absence of inequality and a greater absolute value of PAF and PAR indicates a higher level of inequality. PAR is calculated as the difference between the subgroup with the lowest estimate and the national average of the indicator for adverse outcome indicators. For ordered dimensions like wealth and education, PAR is the difference between the most advantaged subgroup and the national average, regardless of the indicator type. PAF is calculated by dividing the PAR by the national average, μ, and multiplying the fraction by 100 (i.e. PAF=[PAR/μ] * 100).

For binary dimensions like residence, D is calculated as the difference between the subgroup with the highest estimate (rural) and the subgroup with the lowest estimate (urban), regardless of the indicator type. For ordered dimensions like wealth and education, it is the difference between the most disadvantaged subgroup and the most advantaged subgroup. For binary dimensions like residence, R is calculated as the difference between the subgroup with the highest estimate (rural) and the subgroup with the lowest estimate (urban), regardless of the indicator type. For ordered dimensions like wealth and education, it is the ratio between the most disadvantaged subgroup and the most advantaged subgroup. In the absence of inequality, D and R become 0 and 1, respectively.

Point estimates were calculated and presented with corresponding 95% confidence intervals (CIs). To examine whether SBA shows statistically significant disparities across the subgroups of each inequality stratifier, and to determine whether or not the inequality changed with time, we computed 95% CIs around point estimates of each measure for each survey wave. For all inequality measures other than R, the lower and upper bounds of the CI must not include zero to indicate that inequality exists. We assessed the trend of inequality for each summary measure by referring to the uncertainty intervals (UIs) for the different surveys. When the UIs do not overlap, it implies that there is a statistically significant difference between the two UIs. If the UIs overlap, then no inequality exists. A PAF or PAR of zero means no inequality, while a higher value indicates a relatively higher inequality. The variation in SBA over the period was explored by referring to the 95% UIs of the survey years. When there is an overlap in the UI, it means that there is no statistically significant difference between the UIs and vice versa.

### Ethical approval

This study used publicly available data stored in the HEAT software application. Informed consent for the Ghana DHS was obtained from participants prior to the survey. The DHS Program follows ethical standards for ensuring the protection of respondents’ privacy. ICF International ensures that the survey complies with the U.S. Department of Health and Human Services regulations respecting the rights of human subjects. No further approval was required for this study since the data are secondary and available in the public domain. More details about DHS data and ethical standards are available at https://bit.ly/2XHjJsR.

## Results

### Trends in SBA by different inequality dimensions, 1993–2014

**Figure 1.**
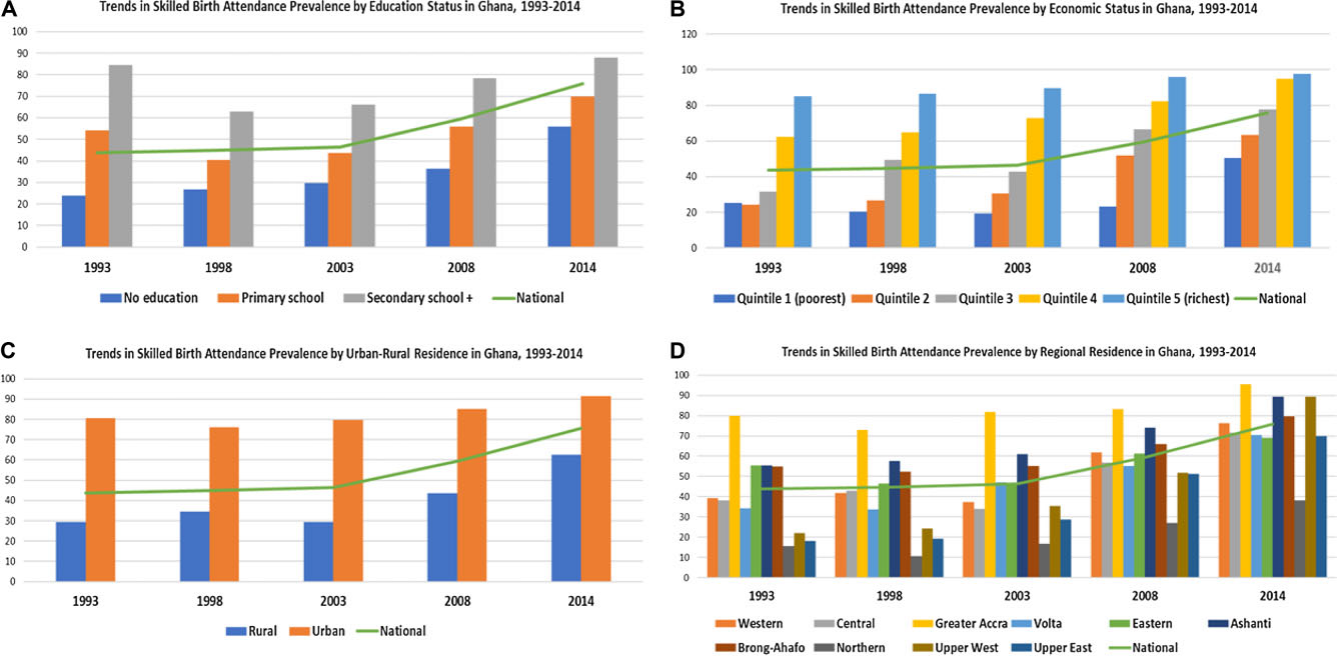
Trends in SBA stratified by education, economic status, urban-rural, and regional residence, 1993–2014.

As shown in Table 1, at the national level, SBA increased marginally between 1993 and 2008. For example, in 1994, SBA was 43.74%, which increased slightly to 44.83% in 1998 and further increased to 46.44% in 2003. However, there was an appreciable increase from 2008 (59.53%) to 2014 (75.81%). Throughout the surveys, SBA was highest among women in the highest wealth quintile and those with secondary or more education. Women in the fourth and fifth wealth quantiles have consistently recorded SBA prevalence higher than the national average, whereas women in the poorest wealth quantile consistently recorded SBA prevalence lower than the national average (Table 1; Figure 1). In all the surveys, women with no formal education recorded SBA prevalence lower than the national average, whereas women with at least a secondary education recorded SBA prevalence higher than the national average (Table 1; Figure 1). The analysis also indicated that SBA was highly concentrated among urban residents in 1993 (80.78 [95% UI 76.20–84.66]) and persisted to 2014 (91.55 [95% UI 88.80–93.68]). Throughout the survey years, rural women recorded SBA prevalence lower than the national average while urban women recorded SBA prevalence higher than the national average (Table 1; Figure 1). With regards to region of residence, the Northern region had the lowest SBA in 2014 (38.21 [95% UI 27.44–50.27]) and the Greater Accra region had the highest (95.41 [95% UI 91.24–97.65]) (Table 1; Figure 1). Women in the Greater Accra, Ashanti and Brong Ahafo regions had SBA prevalence higher than the national average from 1993 through 2014 (Table 1; Figure 1).

**Table 1.** Trends in the prevalence of births attended by skilled health personnel by different inequality dimensions, 1993–2014

	DHS year									
Dimension	n	1993 (N=2204 [43.74%]), % (UI)	n	1998 (N=1925 [44.83%]), % (UI)	n	2003 (N=2171 [46.44%]), % (UI)	n	2008 (N=1757 [59.53%]), % (UI)	n	2014 (N=3507 [75.81%]), % (UI)]
Economic status (quintile)										
1 (poorest)	443	25.28 (19.95–31.49)	513	20.17 (16.48–24.45)	548	19.43 (15.95–23.47)	444	23.07 (19.24–27.40)	769	50.57 (44.01–57.11)
2	489	24.13 (19.79–29.07)	395	26.74 (22.00–32.07)	476	30.39 (25.12–36.23)	380	51.87 (45.56–58.12)	737	63.47 (58.68–68.02)
3	465	31.61 (26.64–37.05)	410	49.29 (43.12–55.48)	449	42.92 (37.51–48.52)	342	66.40 (60.88–71.51)	676	77.85 (72.25–82.59)
4	440	62.27 (56.29–67.90)	341	64.84 (58.04–71.09)	363	72.70 (66.57–78.07)	346	82.21 (76.96–86.47)	680	95.04 (92.28–96.85)
5 (richest)	367	85.29 (80.59–89.00)	266	86.69 (80.37–91.20)	335	89.56 (84.28–93.22)	245	95.80 (91.50–97.97)	645	97.64 (95.64–98.74)
Education										
No education	876	23.86 (20.48–27.60)	710	26.76 (23.13–30.74)	863	29.70 (26.29–33.35)	550	36.46 (31.52–41.69)	926	55.75 (49.41–61.90)
Primary school	1206	54.06 (50.28–57.80)	404	40.47 (34.16–47.12)	511	43.70 (38.18–49.38)	448	55.82 (49.66–61.81)	698	69.79 (65.10–74.11)
≥Secondary school	122	84.43 (75.24–90.63)	811	62.84 (58.30–67.16)	800	66.23 (61.85–70.34)	761	78.39 (74.48–81.84)	1884	87.91 (85.21–90.18)
Place of residence										
Rural	1590	29.43 (25.95–33.18)	1448	34.54 (31.21–38.03)	1442	29.52 (26.48–32.76)	1083	43.54 (39.32–47.85)	1908	62.61 (58.30–66.73)
Urban	614	80.78 (76.20–84.66)	478	76.00 (70.45–80.79)	732	79.73 (75.56–83.35)	676	85.12 (81.32–88.26)	1600	91.55 (88.80–93.68)
Region										
1 Western	206	39.32 (29.95–49.55)	235	41.75 (32.20–51.96)	214	37.35 (30.38–44.90)	170	61.84 (51.90–70.89)	345	76.41 (68.91–82.56)
2 Central	231	38.10 (28.22–49.06)	227	42.91 (35.32–50.85)	184	34.03 (24.56–44.97)	193	56.75 (46.22–66.70)	388	71.54 (65.81–76.65)
3 Greater Accra	199	79.90 (73.90–84.80)	211	72.84 (64.57–79.78)	228	81.72 (73.65–87.73)	201	83.23 (75.90–88.67)	522	95.41 (91.24–97.65)
4 Volta	239	34.31 (24.87–45.18)	202	33.79 (26.49–41.95)	183	47.09 (35.70–58.78)	146	55.02 (44.03–65.54)	278	70.53 (59.21–79.78)
5 Eastern	238	55.46 (46.34–64.23)	266	46.46 (37.16–56.02)	219	46.14 (38.96–53.48)	155	61.31 (52.03–69.84)	327	68.95 (61.70–75.37)
6 Ashanti	400	55.50 (47.79–62.95)	332	57.72 (48.99–65.99)	407	61.09 (54.99–66.86)	323	74.12 (65.70–81.07)	658	89.25 (84.73–92.54)
7 Brong-Ahafo	211	54.98 (45.22–64.37)	154	52.47 (43.72–61.07)	239	55.12 (47.82–62.21)	155	66.01 (55.14–75.42)	314	79.59 (69.46–86.98)
8 Northern	255	15.69 (11.20–21.54)	128	10.69 (6.66–16.73)	297	16.63 (11.02–24.31)	273	27.08 (21.13–33.98)	437	38.21 (27.44–50.27)
9 Upper West	77	22.08 (7.13–51.10)	59	24.19 (18.27–31.28)	69	35.50 (24.33–48.51)	87	51.81 (38.82–64.56)	142	89.34 (85.54–92.23)
10 Upper East	148	18.24 (10.80–29.14)	109	19.11 (13.22–26.81)	129	28.61 (20.76–38.02)	52	51.11 (40.75–61.37)	94	69.76 (56.51–80.37)

### Inequality indices estimates of the factors associated with SBA, 1993–2014

In Table 2, we present the findings from both the absolute (D, PAR) and relative (R, PAF) summary measures, showing that economic inequity existed during the study period (1993–2014). Concerning the simple measures, D and relative R economic inequity in SBA persisted throughout the period. The PAR revealed an increasing inequity trend from 1993 to 2014 while PAF showed a decrease. A significant absolute and relative education-related inequality existed from 1993 to 2014 to the advantage of women with a high level of education, as revealed by all four summary measures. For instance, in 2014, the R and PAR measures of 1.93% (95% UI 1.68–2.18) and 21.83% (95% UI 18.71–24.95) correspondingly revealed significant inequality in SBA to the disadvantage of women with no formal education. The findings also showed substantial rural–urban inequality in favour of urban residents throughout the study period. In 1993, both the complex absolute measure (PAR 37.08 [95% UI 35.60–38.49]) and relative measure (PAF 84.69 [95% CI 81.4–88.0]) showed a decreased magnitude of SBA. In 2014, the complex absolute measure (D, PAR) revealed significant inequality in SBA as revealed by the PAR (15.74 [95% UI 14.38–17.10]).

**Table 2.** Inequality indices estimates of the factors associated with prevalence of births attended by skilled health personnel, 1993–2014

	1993			1998			2003			2008			2014		
Inequality dimension	Est.	LB	UB	Est.	LB	UB	Est.	LB	UB	Est.	LB	UB	Est.	LB	UB
Economic status															
D	60.00	52.91	67.10	66.52	59.89	73.15	70.13	64.38	75.88	72.73	67.67	77.80	47.07	40.34	53.80
PAF	94.99	86.40	103.58	93.35	86.22	100.49	92.88	86.24	99.51	60.92	54.81	67.03	28.79	24.68	32.91
PAR	41.55	37.79	45.30	41.85	38.65	45.05	43.13	40.05	46.21	36.27	32.63	39.91	21.83	18.71	24.95
R	3.37	2.59	4.16	4.30	3.41	5.18	4.61	3.69	5.52	4.15	3.41	4.90	1.93	1.68	2.18
Education															
D	60.57	52.21	68.92	36.08	30.26	41.89	36.53	31.03	42.03	41.93	35.66	48.20	32.17	25.44	38.90
PAF	93.02	87.51	98.53	40.15	33.96	46.35	42.62	37.18	48.07	31.67	25.86	37.49	15.96	12.39	19.54
PAR	40.69	38.28	43.10	18.00	15.23	20.78	19.79	17.27	22.32	18.86	15.39	22.32	12.10	9.39	14.81
R	3.54	2.92	4.15	2.35	1.98	2.72	2.23	1.93	2.53	2.15	1.83	2.47	1.58	1.39	1.76
Place of residence															
D	51.35	45.82	56.88	41.46	35.30	47.63	50.21	45.23	55.19	41.58	36.10	47.07	28.94	24.09	33.80
PAF	84.69	81.39	88.00	69.52	66.38	72.65	71.70	68.20	75.21	42.99	39.77	46.21	20.76	18.97	22.56
PAR	37.04	35.60	38.49	31.17	29.76	32.57	33.30	31.67	34.92	25.59	23.67	27.51	15.74	14.38	17.10
R	2.74	2.38	3.11	2.20	1.94	2.46	2.70	2.39	3.02	1.96	1.75	2.16	1.46	1.36	1.57
Region															
D	64.21	56.76	71.66	62.14	53.10	71.19	65.09	55.51	74.67	56.15	47.13	65.17	57.21	45.24	69.17
PAF	82.67	72.63	92.72	62.46	50.17	74.74	75.98	67.08	84.87	39.82	31.38	48.25	25.85	20.14	31.56
PAR	36.16	31.77	40.55	28.00	22.50	33.51	35.28	31.15	39.41	23.70	18.68	28.72	19.60	15.27	23.92
R	5.09	3.40	6.79	6.81	3.60	10.02	4.91	2.93	6.90	3.07	2.31	3.84	2.50	1.74	3.26

Est, estimate; LB, lower bound; UB, upper bound.

In Table 2, the results show that the most important inequality stratifier of SBA is that of economic status until 2014, when the region of residence showed the strongest effect. More specifically, we observed that the effect of region of residence on SBA was greater than the effects of the other inequality stratifiers. For instance, in 2014, the coefficients of region of residence (D=57.21, PAR=25.85, PAF=19.60, R=2.50) were generally larger than the coefficients of economic status (D=47.07, PAR=28.79, PAF=21.83, R=1.93), education status (D=32.17, PAR=15.96, PAF=12.10, R=1.58) and place of residence (D=28.94, PAR=20.76, PAF=15.74, R=1.46). This result corroborates the estimated prevalence reported in Table 1. As shown in Table 1, the Northern region is the most deprived in terms of SBA. In 2014, as reported earlier, the estimated SBA prevalence in the Northern region was 38.21% compared with SBA estimates for other regions ranging from 69% to 96%.

## Discussion

This study sought to investigate the trends and magnitude of inequalities in the prevalence of births attended by skilled health personnel in Ghana between 1993 and 2014. Our study revealed that SBA increased marginally from 1993 to 2008 and experienced a substantial increase between 2008 and 2014. This variation in magnitude can be attributed to financial and policy issues. Before 2008, out-of-pocket payment for maternal health services in Ghana posed significant barriers for some women and discouraged utilization of SBA.^24^ However, with the introduction of the free maternal healthcare policy in 2008, this burden on women decreased significantly.^15,24–26^ Plausibly, the introduction of free maternal healthcare in 2008 removed some financial barriers and made SBA services more accessible for all women, irrespective of their socio-economic status.

Findings from this study indicate that utilization of SBA is highly concentrated among women with secondary or higher education and significantly decreased for women with either a primary education or no formal education at all. This finding corroborates the findings of Budu,^27^ who found that the likelihood to opt for home birth or an unskilled birth attendant decreased as the level of education of the woman increased. Women with no or primary education tend to opt for home delivery without the assistance of a skilled birth attendant primarily due to their dearth of knowledge and information about the repercussions of home deliveries without skilled assistance.^27^ Likewise, other studies^28,29^ have reiterated that highly educated women are more likely to utilize SBA during delivery. This is probably due to high decision-making autonomy enjoyed by women with higher levels of education.^30^ Also, educated women may easily access health information, especially on the benefits of using SBA, and this may inform their decision to utilize SBA services. In Ghana, women make the decisions regarding where to give birth, thus the education level of mothers plays an important role.^27^

The study also revealed trends in wealth-related inequality in SBA utilization in Ghana. Consistent with findings from previous studies,^28,31–33^ our study found that women in the richest wealth quintile mostly give birth with skilled assistance compared with their counterparts in the poorest wealth quintile. This finding is worrying, but it is expected that the introduction of policies like free maternal health care will translate into more women in the poorest wealth quintile having the opportunity to access and utilize SBA. However, there have been some challenges in the implementation of the 2008 free maternal health policy and the National Health Insurance Scheme, as it has been reported that women in Ghana still pay out of pocket for some maternal health services as well as illegal fees.^27^ Although maternal health services are meant to be free, there are reports of illegal fees being charged, and this could pose as a barrier to many women in accessing SBA services.^14,27^ Such practices inhibit women within the poorest wealth quintile from accessing and utilizing SBA, which exacerbates the inequality gap that exists between the richest and poorest women when it comes to accessing SBA services in Ghana.

Additionally, findings from this study showed significant inequalities in the urban–rural dichotomy. From the findings, the prevalence of SBA utilization was higher among women living in urban Ghana. The urban–rural disparities in the prevalence of SBA may be plausibly linked to the availability and accessibility of health facilities for women in urban areas vs those in rural areas.^34^ A woman living in a rural community may be discouraged to go through the struggle of commuting to the nearest health centre, which could be several kilometres away. Also, there are reports that skilled birth attendants in Ghana are unwilling to be posted to rural areas after their training.^29^ This has resulted in the overconcentration of skilled birth attendants in urban areas at the expense of rural areas. A previous study in Ghana that reported a similar finding has cautioned that if this rural–urban disparity is left unabated, it will impede Ghana's effort to significantly reduce maternal and neonatal mortality by 2030.^35^ Similar observations have been reported in Nigeria,^31,36^ India,^37^ Cambodia^38^ and developing countries as a whole.^39^

Finally, the study showed that the use of SBA was unequal across regions in Ghana. The trend indicates significant levels of inequalities with regard to the regional proportion of women who had births with SBA, with the Northern region and Greater Accra region recording the lowest and highest SBA, respectively, in 2014. This finding could be because the Northern region is predominantly rural, characterized by dispersed settlements, and also the region being one of the poorest in Ghana compared with the Greater Accra region, which is predominantly urban and also the administrative capital of Ghana.^16^ In the northern part of Ghana, unlike the Upper East region which has the highest likelihood of SBA due to the proliferation of community-based health planning and services within the region,^28^ the Northern region has inadequate health services and centres and this has encouraged home deliveries without SBA.^27^ In support of our findings, Budu^27^ has reported that the Northern region has more women giving birth at home, where SBA is likely to be absent. Our finding could also be attributed to the reported shortage of skilled birth attendants in the Northern, Upper West, and Upper East regions.^40^ A shortage of skilled birth attendants means that women in these regions cannot access SBA services even if they want to or they have to either travel longer distances or be in longer queues to access these services. This becomes a disincentive for SBA use in these regions.

## Limitations of the study

This study followed a repeated cross-sectional study design and, as such, causal inference cannot be made. The first version of the GDHS was not included in this study because some of the variables were not consistent with the surveys conducted from 1993 to 2014. Also, there is a possibility of reporting bias introduced by women with more than one child which could originate from misreporting of the child’s age or under-/overreporting of children 2–3 y of age. Despite these limitations, the study provides a nationally representative coverage of SBA and hence findings and recommendations are generalizable to all women 15–49 y of age in Ghana.

### Conclusions

Ghana is determined to significantly reduce maternal and neonatal mortality through SBA. As such, there has been growing interest in research to facilitate and inform policies and interventions towards the achievement of SDG 3.1 and 3.2. To this end, we investigated the trends of inequalities in the prevalence of births attended by skilled health personnel in Ghana. The study concludes that there are significant inequalities in births attended by skilled personnel in Ghana across the dimensions of education level, wealth, place of residence, and region since 1993. Therefore, to enhance skilled birth deliveries, there is the need for policymakers and interventionists to design and develop targeted policies and programs that are tailored to the needs of the subpopulations at risk, i.e. women with no formal education, those within the poorest wealth quintile, rural-dwelling women, and women in the Northern region. This will facilitate the uptake of SBA services and ultimately translate into the realization of SDG 3.1 and 3.2 by 2030.

Further studies will be needed to ascertain the level of regional inequalities in SBA to focus on the context-specific setbacks as well as interventions for these regions. Also, the fact that women in the poorest wealth quintile had the fewest SBA deliveries infers that the national health insurance scheme (NHIS) is not pro-poor enough, thereby leading to out-of-pocket payments that defeat the purpose of the 2008 free maternal healthcare policy and the NHIS. Therefore it is imperative for the government to review the free maternal healthcare policy and ensure that it is absolutely free, especially for women in the poorest wealth quintile. Perhaps NHIS subscription fees and some minor payments can be subsidized or absorbed by the government to ensure easy financial access.

## Data Availability

The dataset can be accessed at https://dhsprogram.com/data/available-datasets.cfm.

## References

[1] Hogan MC , Foreman KJ, Naghavi M et al. Maternal mortality for 181 countries, 1980–2008: a systematic analysis of progress towards Millennium Development Goal 5. Lancet. 2010;375(9726):1609–23. 20382417 10.1016/S0140-6736(10)60518-1

[2] Lozano R , Wang H, Foreman KJ et al. Progress towards Millennium Development Goals 4 and 5 on maternal and child mortality: an updated systematic analysis. Lancet. 2011;378(9797):1139–65. 21937100 10.1016/S0140-6736(11)61337-8

[3] Crowe S , Utley M, Costello A et al. How many births in sub-Saharan Africa and South Asia will not be attended by a skilled birth attendant between 2011 and 2015? BMC Pregnancy Childbirth. 2012;12(1):4. 22251749 10.1186/1471-2393-12-4 PMC3274439

[4] Waniala I , Nakiseka S, Nambi W et al. Prevalence, indications, and community perceptions of caesarean section delivery in Ngora District, Eastern Uganda: mixed method study. Obstet Gynecol Int. 2020;2020:5036260. 32765611 10.1155/2020/5036260 PMC7387994

[5] World Health Organization . 2018 definition of competent maternal and new born health professionals providing care during childbirth. Available from: https://www.who.int/news/item/12-06-2018-definition-of-competent-maternal-and-newborn-health-professionals-providing-care-during-childbirth [accessed 29 October 2022].

[6] Asamoah BO , Agardh A, Cromley EK. Spatial analysis of skilled birth attendant utilization in Ghana. Glob J Health Sci. 2014;6(4):117–27. 10.5539/gjhs.v6n4p117 PMC4825352 24999146

[7] Adatara P , Strumpher J, Ricks E et al. Cultural beliefs and practices of women influencing home births in rural Northern Ghana. Int J Womens Health. 2019;11:353–61. 31239788 10.2147/IJWH.S190402 PMC6556529

[8] World Health Organization . Tracking universal health coverage: first global monitoring report. Geneva: World Health Organization; 2015.

[9] Barros AJ , Ronsmans C, Axelson H et al. Equity in maternal, newborn, and child health interventions in countdown to 2015: a retrospective review of survey data from 54 countries. Lancet. 2012;379(9822):1225–33. 22464386 10.1016/S0140-6736(12)60113-5

[10] World Health Organization . State of inequality: reproductive, maternal, newborn and child health. Geneva: World Health Organization; 2015.

[11] United Nations Population Fund . Giving birth should not be a matter of life and death. New York: United Nations Population Fund; 2016.

[12] World Health Organization . World health statistics 2010. Geneva: World Health Organization; 2010.

[13] World Health Organization . Maternal mortality fact sheet. Geneva: World Health Organization; 2016.

[14] Ameyaw EK , Dickson KS. Skilled birth attendance in Sierra Leone, Niger, and Mali: analysis of demographic and health surveys. BMC Public Health. 2020;20:164. 32013896 10.1186/s12889-020-8258-z PMC6998232

[15] Witter S , Adjei S, Armar-Klemesu M et al. Providing free maternal health care: ten lessons from an evaluation of the national delivery exemption policy in Ghana. Glob Health Action. 2009;2(1):1881. 10.3402/gha.v2i0.1881 PMC2779941 20027275

[16] Ghana Statistical Service, Ghana Health Service, ICF International . Ghana Demographic and Health Survey 2014. Rockville, MD: ICF International; 2015.

[17] Ghana Statistical Service . Multiple indicator cluster survey, 2011: monitoring the situation of children, women, and men; with an enhanced malaria module and biomarker. Accra: Ghana Statistical Service; 2012.

[18] Woldeamanuel BT , Aga MA. Trends, regional inequalities and determinants in the utilization of prenatal care and skilled birth attendant in Ethiopia: a multilevel analysis. Clin Epidemiol Glob Health. 2021;11:100771.

[19] Saxena D , Vangani R, Mavalankar D et al. Inequity in maternal health care service utilization in Gujarat: analyses of district-level health survey data. Glob Health Action. 2013;6(1):19652. 10.3402/gha.v6i0.19652 PMC3591509 23469890

[20] Tsegay Y , Gebrehiwot T, Goicolea I et al. Determinants of antenatal and delivery care utilization in Tigray region, Ethiopia: a cross-sectional study. Int J Equity Health. 2013;12:30. 23672203 10.1186/1475-9276-12-30 PMC3658893

[21] Ghana Statistical Service . 2010 population & housing census: regional analytical report. Accra: Ghana Statistical Service; 2013.

[22] Rutstein SO , Johnson K. The DHS wealth index. DHS comparative reports no. 6. Calverton, MD: ORC Macro; 2004.

[23] Hosseinpoor AR , Nambiar D, Schlotheuber A et al. Health Equity Assessment Toolkit (HEAT): software for exploring and comparing health inequalities in countries. BMC Med Res Method. 2016;16(1):141. 10.1186/s12874-016-0229-9 PMC5069829 27760520

[24] Bosomprah S , Aryeetey GC, Nonvignon J et al. A decomposition analysis of change in skilled birth attendants, 2003 to 2008, Ghana demographic and health surveys. BMC Pregnancy Childbirth. 2014;14:415. 25547321 10.1186/s12884-014-0415-x PMC4314742

[25] Anafi P , Mprah WK, Jackson AM et al. Implementation of fee-free maternal health-care policy in Ghana: perspectives of users of antenatal and delivery care services from public health-care facilities in Accra. Int Q Community Health Education. 2018;38(4):259–67. 29523057 10.1177/0272684X18763378

[26] Opoku E. Utilization of maternal care services in Ghana by region after the implementation of the free maternal care policy. Master's thesis, University of North Texas, 2009.

[27] Budu E. Predictors of home births among rural women in Ghana: analysis of data from the 2014 Ghana Demographic and Health Survey. BMC Pregnancy Childbirth. 2020;20(1):523. 32912164 10.1186/s12884-020-03211-4 PMC7488046

[28] Ameyaw EK , Tanle A, Kissah-Korsah K et al. Women's health decision-making autonomy and skilled birth attendance in Ghana. Int J Reprod Med. 2016;2016:6569514. 28116348 10.1155/2016/6569514 PMC5220507

[29] Kwansah J , Dzodzomenyo M, Mutumba M et al. Policy talk: incentives for rural service among nurses in Ghana. Health Policy Plan. 2012;27(8):669–76. 22349086 10.1093/heapol/czs016

[30] Mengesha ZB , Biks GA, Ayele TA. Determinants of skilled attendance for delivery in Northwest Ethiopia: a community based nested case control study. BMC Public Health. 2013;13:130. 23402542 10.1186/1471-2458-13-130 PMC3577480

[31] Osamor PE , Grady C. Women's autonomy in health care decision-making in developing countries: a synthesis of the literature. Int J Womens Health. 2016;8:191–202. 27354830 10.2147/IJWH.S105483 PMC4908934

[32] Dalinjong PA , Wang AY, Homer CS. Has the free maternal health policy eliminated out of pocket payments for maternal health services? Views of women, health providers and insurance managers in Northern Ghana. PLoS One. 2018;13(2):e0184830. 29389995 10.1371/journal.pone.0184830 PMC5794072

[33] Dixon J , Tenkorang EY, Luginaah IN et al. National health insurance scheme enrolment and antenatal care among women in Ghana: is there any relationship? Trop Med Int Health. 2014;19(1):98–106. 24219504 10.1111/tmi.12223

[34] Hotchkiss DR , Godha D, Do M. Expansion in the private sector provision of institutional delivery services and horizontal equity: evidence from Nepal and Bangladesh. Health Policy. 2014;29(Suppl 1):i12–9. 10.1093/heapol/czt062 PMC4095922 25012794

[35] Amoakoh-Coleman M , Ansah EK, Agyepong IA et al. Predictors of skilled attendance at delivery among antenatal clinic attendants in Ghana: a cross-sectional study of population data. BMJ Open. 2015;5(5):e007810. 10.1136/bmjopen-2015-007810 PMC4442247 25991459

[36] Ekele BA , Tunau KA. Place of delivery among women who had antenatal care in a teaching hospital. Acta Obstet Gynecol Scand. 2007;86(5):627–30. 17464595 10.1080/00016340601134622

[37] Nair M , Ariana P, Webster P. What influences the decision to undergo institutional delivery by skilled birth attendants? A cohort study in rural Andhra Pradesh, India. Rural Remote Health 2012;12:2311. 23148477

[38] Yanagisawa S , Oum S, Wakai S. Determinants of skilled birth attendance in rural Cambodia. Trop Med Int Health. 2006;11(2):238–51. 16451349 10.1111/j.1365-3156.2005.01547.x

[39] Stanton C , Blanc AK, Croft T et al. Skilled care at birth in the developing world: progress to date and strategies for expanding coverage. J Biosoc Sci. 2007;39(1):109–20. 16522226 10.1017/S0021932006001271

[40] Rishworth A , Dixon J, Luginaah I et al. “I was on the way to the hospital but delivered in the bush”: maternal health in Ghana's Upper West Region in the context of a traditional birth attendants’ ban. Soc Sci Med. 2016;148:8–17. 26638143 10.1016/j.socscimed.2015.11.018

